# Application of Isotachophoresis to Differentiate Organic Matter in Environmental Samples

**DOI:** 10.3390/molecules30163347

**Published:** 2025-08-12

**Authors:** Przemysław Kosobucki

**Affiliations:** Department of Food Analysis and Environmental Protection, Faculty of Chemical Technology and Engineering, University of Science and Technology of Bydgoszcz, 3 Seminaryjna Street, 85-326 Bydgoszcz, Poland; p.kosobucki@pbs.edu.pl

**Keywords:** humic acids, fulvic acids, isotachophoresis, ecoanalytics

## Abstract

This paper presents the results of the application of isotachophoresis to analyze organic matter in environmental samples (sewage sludge, river water). The results obtained indicate that isotachophoresis can be successfully used to distinguish humic acids from fulvic acids. The proposed isotachophoretic analysis method was optimized in terms of leading and terminating electrolyte composition, operating current, column length, and detection mode using a model mixture of humic acids (Fluka).

## 1. Introduction

Soil organic matter, the largest part of which is soil humus, is a very important soil component for soil functioning. Among others, the soil organic matter content is one of the main indicators of the quality of the soil environment, which is due to the conservative role of soil organic matter in the physical (bulk density, aggregate stability, water holding), physical/chemical (ion exchange, buffer properties, solubility and mobility of elements, detoxification of the environment, the source of macro- and micronutrients for plants) and biological properties of the soil (nutrient and energy supply to microorganisms, biostimulation of plant growth and development, biodiversity regulation) of the soil. Its role in the structure of the soil is essential; thus, it is the main agent in the formation of the proper aggregate structure of the soil that, in turn, positively influences the soil’s ventilation, permeability, water-holding capacity, and compactness. Soil humus, which is the organic matter part, is involved in the sorption of exchange and the regulation of the buffer capacity of the soil. The humic substances, namely the humic and fulvic acids, influence the solubility and the transport of the metal ions; therefore, they can both provide the plants with the elements (micronutrients) that they need and also reduce the availability of the pollutants that are present in the environment. The humic substances have the capacity not only to bind minerals of the soil but also the particles of the organic pollutants, such as the plant protection products, and the pollutants that are generated from the industry to the environment, including polychlorinated biphenyls (PCBs) and polycyclic aromatic hydrocarbons (PAHs) [1].

Among humic substances distinguished are the following:

- Fulvic acids (FAs), which are water-soluble, have a mobile nature and are of low persistence (half-life period 10–15 years).
- Humic acids (HAs), which are soluble in NaOH and alcohol, and precipitate after adding HCl; they are active, have high sorption ability, and have a half-life of more than 1000 years.
- Humines, insoluble, chemically inactive, very persistent [2].

So, in the sixties, the most common ways to identify humic substances were the so-called destructive testing methods, such as oxidation, reduction, and pyrolysis. Non-destructive methods were then developed and have quite a few among them now, such as the elemental analysis of the oxygen- and nitrogen-containing functional groups. The characteristics of humic substances can also be determined by instrumental analytical methods. These include potentiometric and conductometric titration [3], as well as spectral methods, UV (ultraviolet) and VIS (visible light) [4], fluorescent [5], IR (infrared) [6], NMR (nuclear magnetic resonance) [7], and ESR (electron spin resonance) spectroscopy [8]. Other methods such as X-ray diffraction [9], membrane dialysis [10], size-exclusion chromatography (SEC) [11], liquid chromatography (HPLC) [12], field-flow fractionation (FFF) [13], electrophoretic techniques (isotachophoresis—ITP, capillary zone electrophoresis—CZE) [14,15], and mass spectrometry (MS) might also be used [16]. Sometimes, the easiest methods, for example, elemental analysis (CHN), provide very basic information about the composition of the compounds under investigation. Between humic and fulvic acids, peptides, cellulose, sugars, and lignins, huge differences in the content of carbon, hydrogen, oxygen, and nitrogen are observed [17].

Due to the ionogenic nature of water-soluble humic substances, they are characterized by electrophoretic separation methods. The main advantages of electromigration methods include low reagent consumption, very high efficiency, short analysis time, the possibility (although not always) of simultaneous determination of cations and anions, performing analyses in a wide pH range, and generating small amounts of wastewater. Isotachophoresis (ITP) with simple conductivity detection is a fitting and effective method for sensitive determination of anionic and cationic species in environmental samples analysis (waters, wastewaters, sewage sludge, composts) [18,19,20,21,22,23]. There are several electromigration techniques; ITP is one of them. In the ITP, only one type of species bearing the same charge can be separated at one time (either cations or anions). The discontinuous background electrolyte system is chosen such that the anion or the cation of the leading (LE) and the terminating (TE) electrolytes will have higher and lower mobilities, μ, than the analytes of interest [24].

The main goal of this study is to prove that the isotachophoretic technique may be applied to the identification or the preliminary investigation of natural organic matter in environmental samples. Optimization of the isotachophoretic method (leading and terminating electrolytes composition, applied current, length of the column) and detection mode (UV-VIS (wavelength), conductivity) was performed with a model mixture of humic acids (Fluka). Real samples were collected from the Vistula River (water) and from a wastewater treatment plant in Toruń, Poland (sewage sludge).

## 2. Results and Discussion

**Figure 1:**
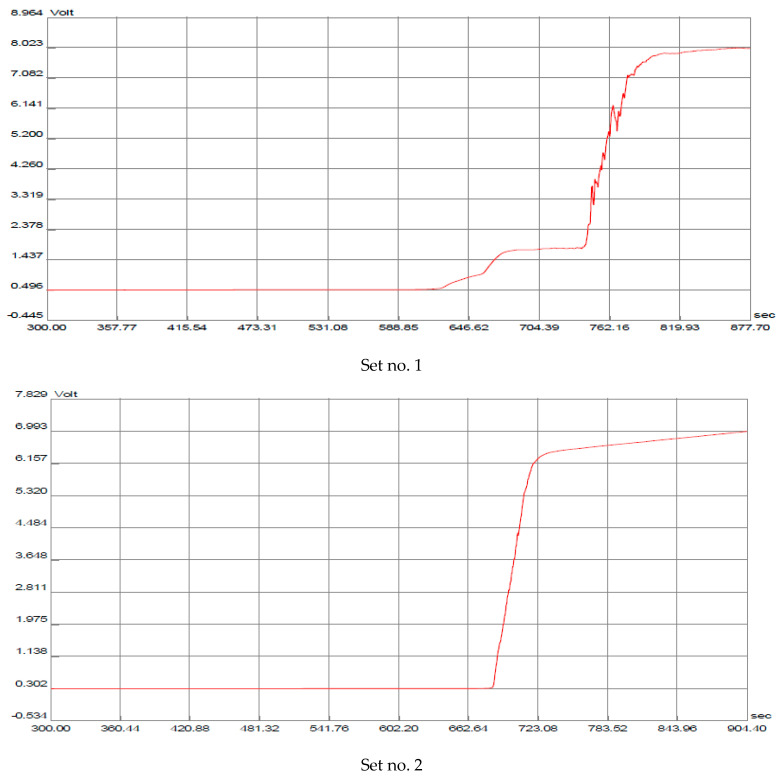

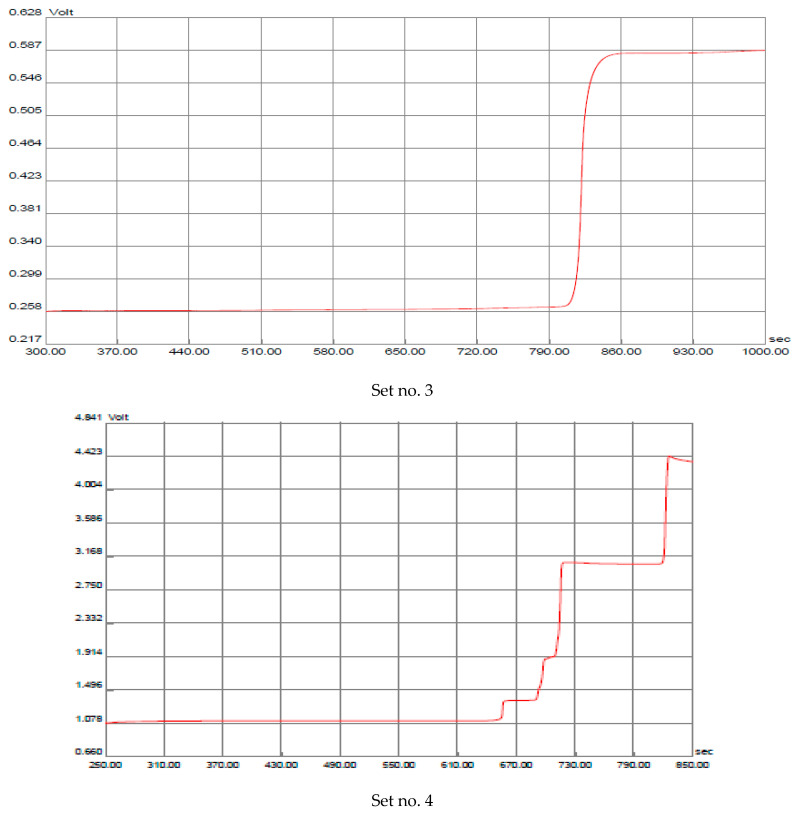
Isotachophoregram from the separation of model humic acids, Fluka, using different electrolyte systems (details see Table 1), migration time (sec) at X-axis and voltage (V) at Y-axis.

The electrolytes employed in the isotachophoretic analyses of the humic acid samples are detailed in Table 1. Detection was performed using conductivity (I_2_ = 100 μA) and UV absorbance measured at 288 nm. The best separations were obtained with the addition of polyvinylpyrrolidone (PVP) in the leading electrolyte. This polymer significantly affects the effective mobilities of particular acids, which are considered the monomeric units of humic materials, as visualized in Figure 1.

The fourth set of electrolytes was selected for investigation based on several considerations. In the proposed electrolyte system, the compounds during analysis are partially ionized and can be separated using zone electrophoresis or isotachophoresis. This is due to the fact that the pKa values of carboxyl groups in formic, acetic, and benzoic acids range from 3.75 to 4.75, while the pKa value of the phenolic OH group in phenol is approximately 10. Carboxyl groups are only partially ionized, while phenolic groups are almost completely non-ionized. Consequently, aggregations of these compounds were anticipated to be negligible during separations performed under conditions favoring acid-base reactions. Moreover, their leading electrolyte (LE) and terminating electrolyte (TE) anions ensured the maximum achievable range in effective mobilities when performing isotachophoretic (ITP) migrations of the humic components at a pH of 3.5. This selection was made in order to maximize the differences in mobility. The reasoning was that chloride, employed as the leading anion, exhibits the highest mobility under these conditions, whereas caproic acids, the terminating anion, display the slowest mobility within anionic ITP separations in aqueous electrolyte systems. Isotachophoregrams 2 and 3 in Figure 1 show that there are no components between the leading and terminating electrolytes. The absence of recorded zones between the LE and TE signals clearly indicates the need to add PVP to the leading electrolyte, which results in a change in the effective mobility of selected acids and enables their separation.

The extraction of humic and fulvic acids from sewage sludge (sourced from the Wastewater Treatment Plant in Toruń, Poland) adhered to the standard alkali extraction method [25].

**Figure 2:**
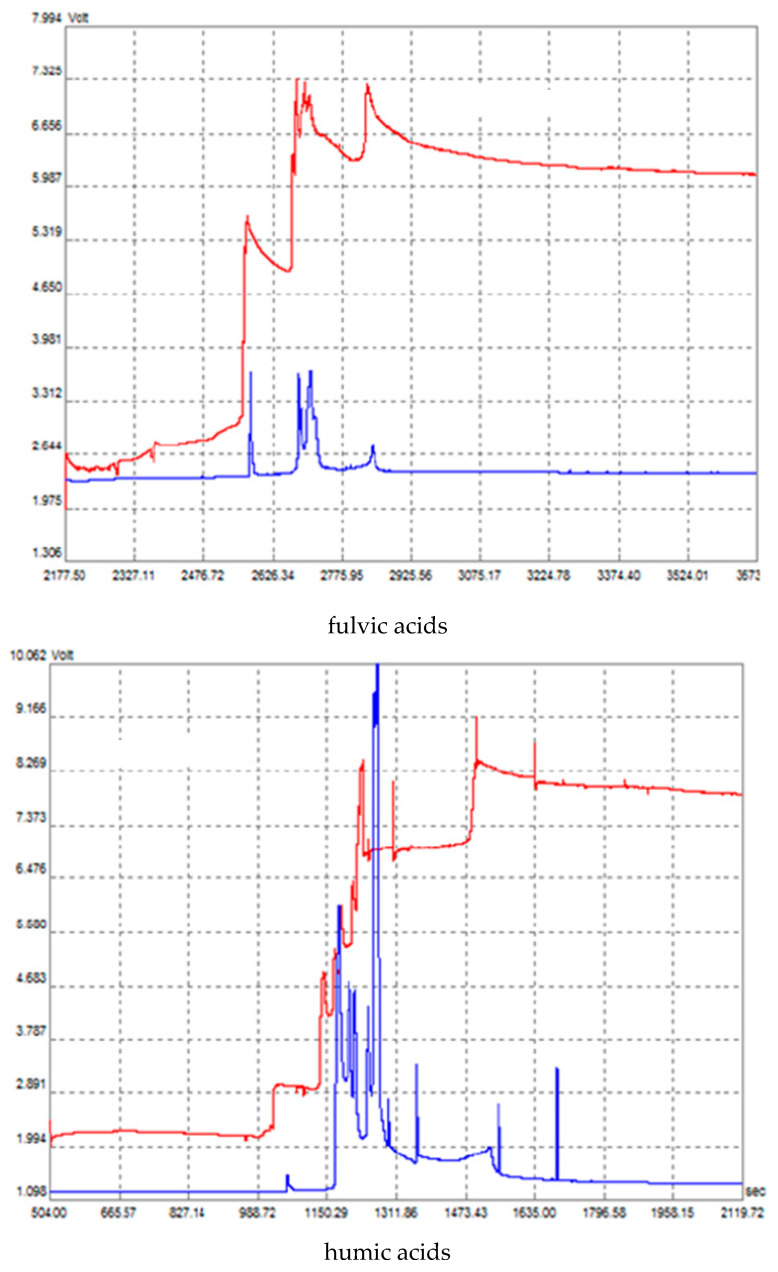
Isotachophoregram from the separation of fulvic acids (FAs) and humic acids (HAs) obtained from sewage sludge using a 4-electrolyte system (red—conductivity detection, blue—UV detection at 288 nm, I_2_—100 μA), migration time (sec) at X-axis and voltage (V) at Y-axis.

Representative isotachophoretic profiles acquired during the separation of sample components, performed using conductivity and UV detection (as depicted in Figure 2), highlighted the sample’s inherent heterogeneity.

The diverse nature revealed by the isotachophoregrams became even more complex due to anionic compounds arising from the leading electrolyte and terminating electrolyte solutions, along with zones of unknown substances. Humic substances, being anionic, migrate with considerable effective mobility within the pH range applied for ITP. Numerous compounds within the samples contribute to the final appearance of the isotachophoregrams. Their spectral characteristics (288 nm, 472 nm, and 664 nm) allow for good selectivity during ITP, leading to the ultimate choice of spectrophotometric detection at 288 nm. However, relying only on UV-VIS detection merely reinforces the heterogeneous nature of humic substances. To make the isotachophoregrams obtained from photometric detection more readily understandable, implementing discrete spacers is required [26].

Given the light absorption properties of humic substances, the existing ITP technique could potentially be refined by employing multi-wavelength photometric detection. Therefore, DAD or dual-wavelength photometric detectors (e.g., using 472 nm and 664 nm to determine the E4/E6 ratio for specific ITP fractions) present alternatives for detection. This leads to more efficient usage of ITP separations during the photometric characterization of humic substance fractions.

## 3. Materials and Methods

### 3.1. Instrumentation

An isotachophoretic analyzer EA 102 (Villa Labeco, Spisska Nova Ves, Slovakia) was used. This analyzer is equipped with two FEP (fluoroethylenepropylene polymer) columns (a pre-separation one: 0.8 mm × 90 mm, and an analytical one: 0.3 mm × 160 mm), two contact conductometric detectors, a UV-VIS detector, and a sample loop of 30 µL. The driving currents were 200 and 40 µA in the preseparation and analytical columns, respectively. The isotachophoretic data were acquired and processed by an ITPPro 32 software (KasComp, Bratislava, Slovakia).

### 3.2. Chemicals

All chemicals were obtained from P.O.Ch. (Gliwice, Poland) or from Sigma-Aldrich (Steinheim, Germany). All chemicals utilized for electrolytes, standard solutions, and sample preparation were pure per analysis (p.p.a.) class. Deionized water was obtained from Milli-Q RG (Millipore, Molsheim, France).

A standard humic acid (HA) with a molecular mass in the range of 600–1000 Da was obtained from Fluka (Buchs, Switzerland). A stock solution (0.2% *w*/*v*) was prepared by dissolving the acid in a 0.001 M aqueous solution of sodium hydroxide. The pH of the solution was adjusted to 5.0 by the addition of morpholinoethanesulfonic acid (MES). A five-fold diluted stock solution was taken for ITP experiments.

## 4. Conclusions

The data indicates that when polyvinylpyrrolidone (PVP) is present in the leading electrolyte (LE), we gain an ability to assess the extent of hydrophobicity and, as a consequence, infer the presence of aromatic structures within humic substances.

Variations in hydrophobicity, molecular masses, and the resulting solubilities of the various components are essential characteristics for distinguishing between humic acids (HAs) and fulvic acids (FAs) using established methodologies. These findings imply that the approach utilized here holds promise as a tool for this differentiation, bringing forth certain clear benefits such as quick analysis, reduced labor demands, and requiring only minimal sample preparation.

The technique of isotachophoresis can function as a screening tool, yielding results that act as a distinctive “fingerprint.”

## Figures and Tables

**Table 1:** Tested operational systems for isotachophoretic separations of humic acids.

	Set no. 1	Set no. 2	Set no. 3	Set no. 4
Solvent	Water	Water	Water	Water
Leading anion (LE)	Cl^-^	Cl^-^	Cl^-^	Cl^-^
Concentration [mM]	10	10	10	10
Counter ion	β-alanine	β-alanine	β-alanine	β-alanine
Additive to the LE	0.1% (*w*/*v*) HEC	0.1% (*w*/*v*) HEC	0.1% (*w*/*v*) HEC	0.1% (*w*/*v*) HEC
Co-additive to the LE	0.2% (*w*/*v*) PVP	-	-	0.2% (*w*/*v*) PVP
pH of the LE	3.5	3.5	3.5	3.5
Terminating anion (TE)	EACA	Caproic acid	EACA	Caproic acid
Concentration [mM]	5	5	10	5

where HEC—hydroxyethylcellulose, PVP—polyvinylpyrrolidone 360,000, EACA—6-aminocaproic acid.

## Data Availability

Data is contained within the article.
